# Functional Properties of Two-Component Hydrogel Systems Based on Gelatin and Polyvinyl Alcohol—Experimental Studies Supported by Computational Analysis

**DOI:** 10.3390/ijms22189909

**Published:** 2021-09-14

**Authors:** Karolina Labus, Lukasz Radosinski, Piotr Kotowski

**Affiliations:** 1 Department of Micro, Nano and Bioprocess Engineering, Faculty of Chemistry, Wrocław University of Science and Technology, Norwida 4/6, 50-373 Wrocław, Poland; 2 Department of Mechanics, Materials and Biomedical Engineering, Faculty of Mechanical Engineering, Wrocław University of Science and Technology, Smoluchowskiego 25, 50-370 Wrocław, Poland; piotr.kotowski@pwr.edu.pl

**Keywords:** biodegradable hydrogels, gelatin, polyvinyl alcohol, functional properties, enzyme carrier, molecular dynamics

## Abstract

The presented research is focused on an investigation of the effect of the addition of polyvinyl alcohol (PVA) to a gelatin-based hydrogel on the functional properties of the resulting material. The main purpose was to experimentally determine and compare the properties of hydrogels differing from the content of PVA in the blend. Subsequently, the utility of these matrices for the production of an immobilized invertase preparation with improved operational stability was examined. We also propose a useful computational tool to predict the properties of the final material depending on the proportions of both components in order to design the feature range of the hydrogel blend desired for a strictly specified immobilization system (of enzyme/carrier type). Based on experimental research, it was found that an increase in the PVA content in gelatin hydrogels contributes to obtaining materials with a visibly higher packaging density, degree of swelling, and water absorption capacity. In the case of hydrolytic degradation and compressive strength, the opposite tendency was observed. The functionality studies of gelatin and gelatin/PVA hydrogels for enzyme immobilization indicate the very promising potential of invertase entrapped in a gelatin/PVA hydrogel matrix as a stable biocatalyst for industrial use. The molecular modeling analysis performed in this work provides qualitative information about the tendencies of the macroscopic parameters observed with the increase in the PVA and insight into the chemical nature of these dependencies.

## 1. Introduction

Biodegradable hydrogels are a specific group of functional materials that are gaining more and more applicability in various fields. The most important ones include medicine [1,2,3], tissue engineering [4,5,6], pharmaceutics [7,8,9], diagnostics [10,11,12], the food industry [13,14,15], cosmetics [16], and gardening and agriculture [17,18,19,20]. Despite their biodegradability, the greatest merits of such hydrogels are their biocompatibility, semi-permeability, ability to create multilayer systems, and maintenance of the spatial structure by retaining aqueous solutions in the interchain voids [1,21,22,23,24]. Knowledge of the characteristic properties of these biomaterials (such as the degree of cross-linking, the porosity, and the swelling ability) is essential for their effective usage in strictly specified applications.

Currently, one of the most widely studied applications is the use of biodegradable hydrogels as immobilization matrices for various bioactive compounds (e.g., drugs, enzymes, antibodies, nutrients, microelements, and agrochemicals) [20,25,26,27,28]. Depending on the final application: (1) as a system for the controlled release of the active compound or (2) as an immobilized biocatalyst with high stability different properties of hydrogel matrices are required. In the former case, it is important to fine-tune the properties of the material in terms of a suitable permeability that has been adapted to the size of the compound to be released and/or an appropriate change in the swelling ratio at the target area in the response to the specific external stimulus (e.g., temperature, pH, ionic strength, or electric field). In the latter application, the main purpose is to obtain a cross-linked hydrogel matrix that allows for the permanent immobilization of the enzyme and, at the same time, the free exchange of the reactants during the catalytic reaction. Despite these differences, in both cases, it is crucial to possess information on the basic functional features of the hydrogels used and the relationships between them. It should be noted that the properties of these materials can be modified to some extent, predominantly by changing their chemical composition [29,30,31]. Due to the use of different proportions of two or more polymeric building blocks, it is possible to influence the sorption properties (e.g., the swelling ability, the water absorption capacity, and hydrolytic degradation) as well as the mechanical resistance or sensitivity to external stimuli of the resulting material.

Biodegradable hydrogels can be made from a wide range of natural and synthetic polymers as well as blends of these polymers, with the proviso that at least one is hydrophilic in nature [32,33,34,35,36]. Given the multitude of possible combinations, experimental studies on the effect of individual components on the properties of obtained hydrogels require a huge expenditure of work and time and financial support. Therefore, in order to improve the research economics, alternative possibilities for preliminary estimation of material features are sought that would precede the main laboratory tests. In this case, one of the approaches currently under development is the use of suitable computational tools to design spatial models of cross-linked hydrogel structures [37,38] and to determine theoretical values of specific parameters (e.g., the Flory-Huggins interaction parameter, the sorption capacity) that could characterize the real functional properties of materials.

In order to properly perform such a computational analysis of the influence of the chemical structure on a hydrogel’s properties, it is necessary to go through a number of important test stages. First of all, it is crucial to create a molecular model of the given hydrogel, which is quite a challenge, especially for complex systems with two or more components and single-component systems based on polypeptide chains. Then, using the developed computational methodology, it is necessary to determine the structural parameters of the hydrogel network and check the extent to which the received results reflect the values obtained experimentally for the given material properties.

Computational methods provide valuable insight into predicting the properties of the final material depending on the proportions of both components and experimental verification of the suitability of the developed methodology and its relation to the chemical composition on the molecular level. The research presented in this article was focused on an investigation of how the addition of PVA to a gelatin-based hydrogel affects the functional properties of a material intended to be effectively used as a carrier for the immobilization of invertase. This enzyme has a high industrial impact and is widely used in various food processing sectors, such as the beverage, confectionery, bakery and production of invertase sugar, high-fructose syrup, artificial honey, and animal feed sectors [39].

The main purpose of this study was experimental determination and comparison of the properties of materials differing in the content of doped PVA. The utility of these matrices for the production of an immobilized invertase preparation with improved operational stability was examined. We also propose a useful computational tool for predicting the properties of the final material depending on the proportions of both components in order to design the feature range of the hydrogel blend desired for a strictly specified immobilization system (of enzyme/carrier type).

Gelatin and PVA were not chosen by accident. Both of these polymers are biodegradable [8,40,41] and have been approved by the Food and Drug Administration (FDA) as compounds Generally Recognized As Safe (GRAS) [42,43]. This is important in order to ensure that the research is in accordance with current pro-ecological trends. Moreover, considering the principal goal of our study, it is significant that they differ in origin, spatial structure, and gelling properties. Polyvinyl alcohol (PVA) is a water-soluble synthetic polymer composed of repeating units of –CH_2_–CH(OH)– [44,45]. Due to the high number of hydroxyl groups, PVA can be effectively used to form a variety of hydrogels by physical or chemical cross-linking. In this case, the most commonly used methods are freeze-thawing [46] and complexation with borate ions [47]. In contrast, gelatin is a hydrophilic biopolymer of natural origin built with polypeptide chains composed of a wide range of amino acids with diverse side groups [48,49]. It is widely used in the preparation of hydrogels for various applications. Most often, these materials are produced by chemical cross-linking of gelatin with, e.g., genipin or transglutaminase [50,51,52,53,54].

Taking into account the diversity of these two polymers, we assumed that the hydrogel materials obtained by blending them will noticeably differ in their functional properties. Furthermore, we believe that the selection of such a two-component model system makes it possible to perform a detailed analysis on the molecular level of the reasons for changes in the properties of the obtained hydrogel materials.

The research was divided into two separate pathways. On the first path, the properties of the obtained hydrogels (e.g., the pore distribution, the mechanical resistance, the swelling capacity, the water absorption capacity, and hydrolytic degradation) were experimentally determined. We also analyzed how the chemical composition of the tested materials influences their functional properties and their applicability as carriers for enzyme immobilization in the invertase case study. The second path was focused primarily on the theoretical development of molecular models of given hydrogel systems and the determination of the network parameters that characterize the 3D structures of the obtained materials. In the final stage of the research, we examined whether the proposed methodology for computational estimation of the properties of gelatin/PVA-based hydrogel materials reflects with good probability the trends obtained experimentally.

## 2. Results

### 2.1. Experimental Studies on Functional Properties of Gelatin and Gelatin/PVA Hydrogels

The main aim of the experimental study was to determine the effect of the addition of polyvinyl alcohol on the functional properties of the gelatin-based hydrogel. For this purpose, three materials with different chemical compositions were considered. Briefly, they were composed of: (1) 10% *w*/*v* of gelatin and 0% *w*/*v* of PVA (G/PVA 0%); (2) 10% *w*/*v* of gelatin and 1% *w*/*v* of PVA (G/PVA 1%); and (3) 10% *w*/*v* of gelatin and 2% *w*/*v* of PVA (G/PVA 2%). During the research, the structural, mechanical, and sorption properties and the ability of the tested materials to be matrices for enzyme immobilization were determined.

#### 2.1.1. Structural Properties of Gelatin and Gelatin/PVA Hydrogels

**Figure 1:**
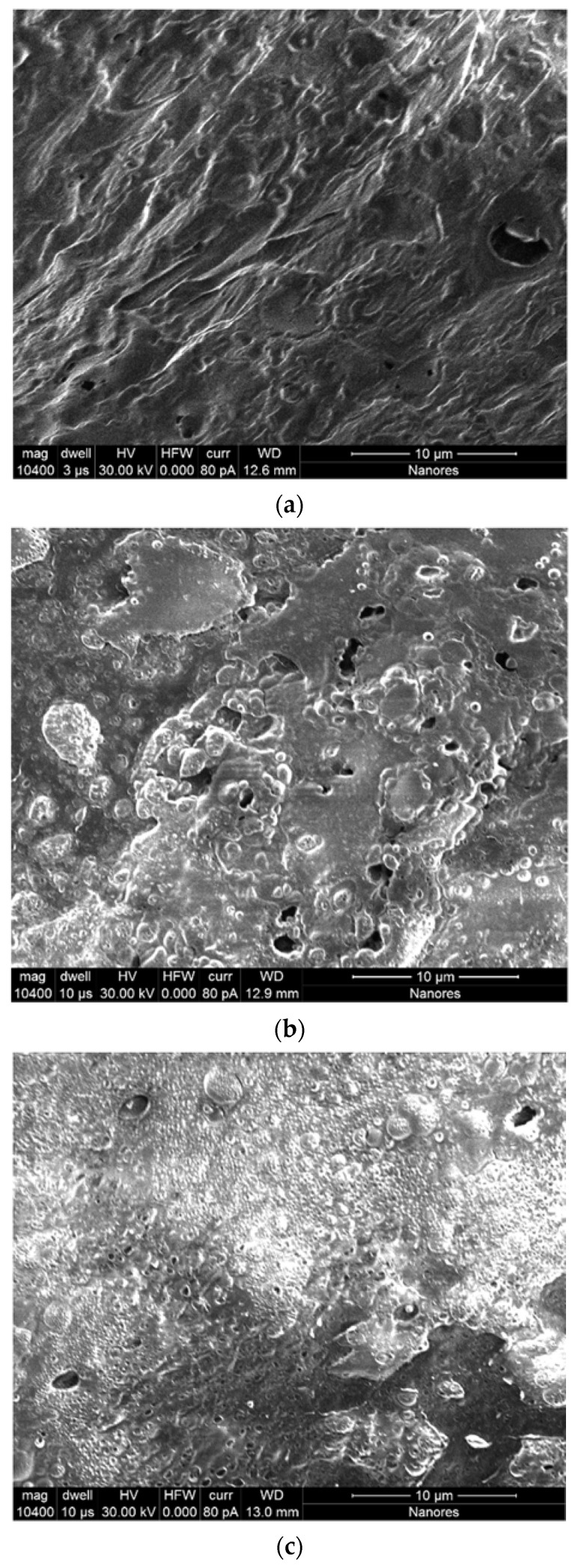
Sample SEM images of hydrogel materials based on gelatin (G) and polyvinyl alcohol (PVA). Gelatin-based hydrogel without PVA (G/PVA 0%) (**a**); gelatin-based hydrogel with 1% *w*/*v* of PVA (G/PVA 1%) (**b**); and gelatin-based hydrogel with 2% *w*/*v* of PVA (G/PVA 2%) (**c**). In each case, the concentration of gelatin was 10% *w*/*v*.

Firstly, SEM imaging of the tested hydrogel materials was performed. The results of the microscopic measurements are depicted in Figure 1. As can be observed, all of the materials present an amorphous structure. Nevertheless, there can be noticed some differences between the hydrogels subjected to micro-imaging. Preliminary analysis of sample SEM images indicates that as the PVA content in the tested hydrogel blends increased, the pore size in the structure of these materials visibly decreased.

**Figure 2:**
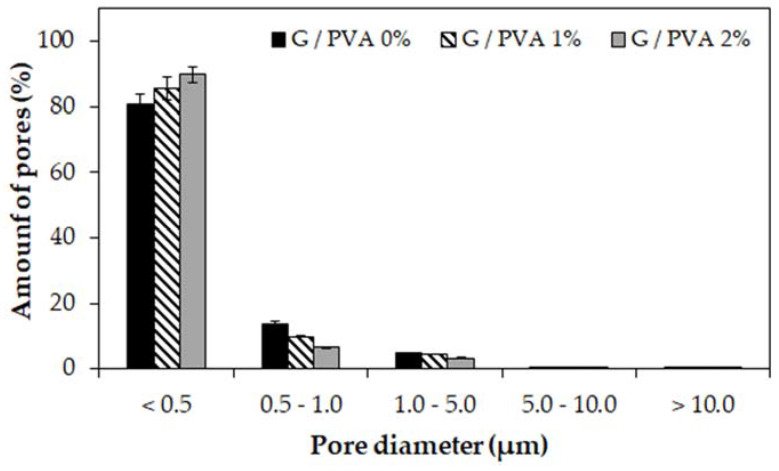
Histograms of the pore diameter distribution in hydrogel materials based on gelatin (G) and polyvinyl alcohol (PVA). Gelatin-based hydrogel without PVA (G/PVA 0%, black), a gelatin-based hydrogel with 1% *w*/*v* of PVA (G/PVA 1%, striped), and gelatin-based hydrogel with 2% *w*/*v* of PVA (G/PVA 2%, grey). In each case, the concentration of gelatin was 10% *w*/*v*.

In order to precisely delineate these differences, the pore distribution in the tested hydrogels was determined based on standardized series of SEM micrographs. This detailed analysis was performed for each material sample on 30 such images. As a result, histograms of the pore diameter distribution in each hydrogel varying in the content of PVA were obtained (Figure 2).

It can be observed that, regardless of chemical composition, in all tested hydrogel samples pores of a size smaller than 0.5 microns are predominant and constitute from 81% to 90% of the total number of pores present in the material structure. Moreover, with the increase in the polyvinyl alcohol content in the gelatin-based hydrogels, the number of smaller pores also gradually increased. At the same time, a decrease in the number of larger pores was noticed, particularly those in the size range of 0.5–1.0 µm and 1.0–5.0 µm. We presume that this direction of change in the pore size distribution depending on the increase in PVA content is related to the PVA molecules’ penetration into and partial filling of free voids between gelatin chains. As a result, an increased number of smaller pores are created between the cross-linked polymer chains in the interpenetrating hydrogel network.

#### 2.1.2. Mechanical Properties of Gelatin and Gelatin/PVA Hydrogels

**Figure 3:**
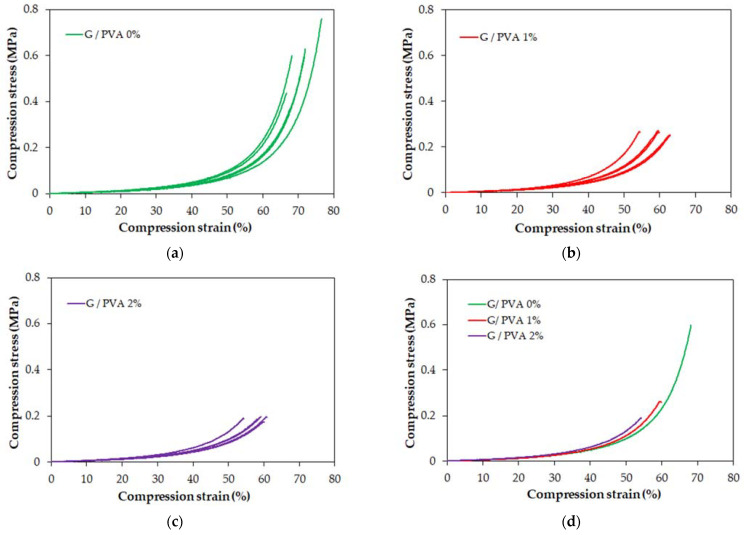
Compression stress-strain curve for a gelatin-based hydrogel without PVA (G/PVA 0%) (**a**), a gelatin-based hydrogel with 1% PVA (G/PVA 1%) (**b**), and a gelatin-based hydrogel with 2% PVA (**c**) and a comparison of the representative stress-strain characteristics for all hydrogels tested (**d**). In each case, the concentration of gelatin was 10% *w*/*v*.

Three types of hydrogels differing in their PVA content were examined. Five samples of a cylindrical shape were prepared for each type of hydrogel. All samples were compressed under the same conditions until failure. The results of the compression in the form of stress-strain graphs are shown in Figure 3.

These graphs show that all tested hydrogels have nonlinear characteristics (i.e., are elastically nonlinear) in the entire range. Therefore, the modulus of elasticity (Young’s modulus) cannot be strictly determined for these materials. Instead, the tangent modulus can be determined for selected strain values. As can be observed in Table 1, this parameter grows with the increase in PVA content in the composition of gelatin/PVA blends. Moreover, for each hydrogel type, the value of the tangent modulus rises straightforwardly with an increase in the strain value specified in the calculation.

The main mechanical property that characterizes hydrogel materials—the failure strength of the material—is reported in Table 2. We observed that the PVA content significantly affected the strength of the hydrogel. The strength is inversely proportional to the content of PVA. On the other hand, it seems that samples containing PVA have a smaller spread of failure strengths.

A comparison of the stress-strain characteristics of the three types of hydrogels is shown in Figure 3d. These characteristics almost coincide, and lower PVA contents allow for higher stress/strain values to be achieved. The maximum strain achieved for all hydrogel samples ranges from 55% to 70%. Presumably, the reduction in compressive strength for the material with increased PVA content may have arisen from the greater number of smaller pores in the hydrogel network (Figure 2), resulting in increased water pressure on the polymer chains that accelerates the material’s rupture.

#### 2.1.3. Water Content in Gelatin and Gelatin/PVA Hydrogels

**Figure 4:**
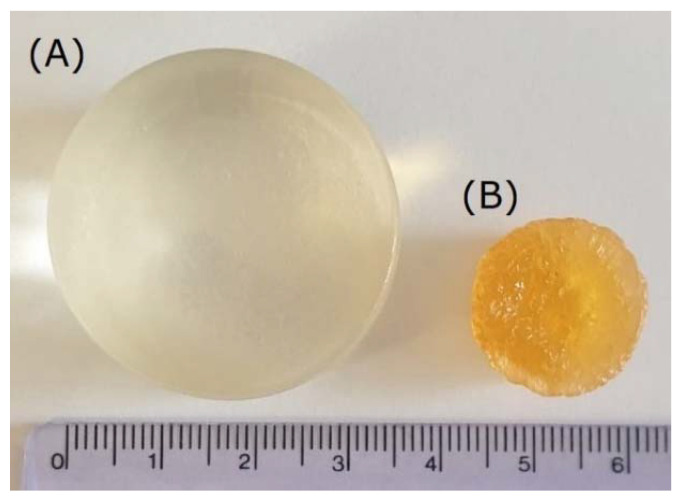
Visual representation of the exemplary gelatin/PVA-based hydrogel material tested in the current study. The material in the hydrated state (**A**) and after drying to a constant weight (**B**).

Figure 4 presents a comparison of an exemplary gelatin/PVA hydrogel in a hydrated and dried form. It can be noticed that the change in diameter is relevant (around 2-fold). Moreover, the difference in volume between the hydrated and dry form is significant as well (approximately 15-fold).

In turn, the differences in the content of the water phase between the cross-linked structure of each tested hydrogel are presented in Table 3.

The obtained results indicate that the water content in the tested materials decreases slightly with the increase in the PVA content. This is most likely related to the increase in packing of the polymer network caused by the creation of interpenetrating gelatin and PVA chains.

#### 2.1.4. Sorption Properties of Gelatin and Gelatin/PVA-Based Hydrogels

The swelling ratio, the water absorption capacity, and the rate of hydrolytic degradation was determined for all types of hydrogels tested. In each case, the values of sorption properties were obtained after 24 h of incubation in a water bath at three different temperatures (30, 40, and 50 °C). The results of the experiments are summarized in Table 4, Table 5 and Table 6.

Regardless of the temperature used, it can be seen that with the increase in the content of PVA, both the swelling degree (Table 4) and the water absorption capacity (Table 5) of the tested materials increased. We relate these phenomena to the increase in the hydrogen-bond donor-acceptor density, which is explained in the next section.

When taking into account the rate of hydrolytic degradation (Table 6), the opposite tendency was observed. In this case, the addition of PVA to the gelatin hydrogel resulted in a noticeable increase in resistance to degradation. Therefore, it can be concluded that the stability of gelatin-based hydrogels increases with increasing PVA content in the material’s composition. In particular, this feature is essential with regard to the practical application of such hydrogel matrices.

#### 2.1.5. Application of Gelatin and Gelatin/PVA Hydrogels as Carriers for Enzyme Immobilization

**Figure 5:**
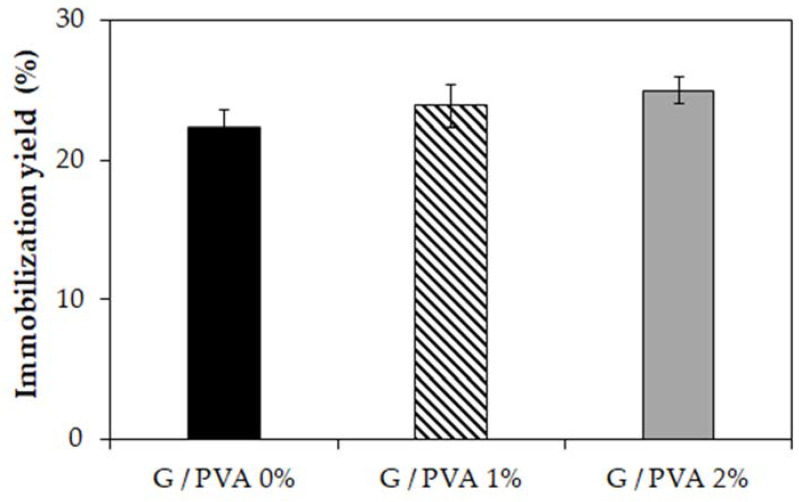
The yield of immobilization of invertase from *Saccharomyces cerevisiae* using different hydrogel matrices as the carrier. Gelatin-based hydrogel without PVA (G/PVA 0%, black), a gelatin-based hydrogel with 1% *w*/*v* of PVA (G/PVA 1%, striped), and gelatin-based hydrogel with 2% *w*/*v* of PVA (G/PVA 2%, grey). Mean value of three independent repetitions.

**Figure 6:**
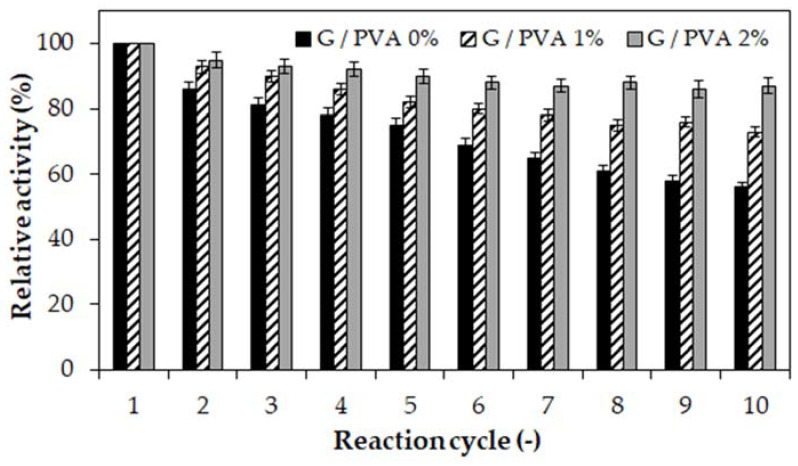
The relative activity of invertase immobilized in different hydrogels was determined after each of 10 consecutive reaction cycles. Enzyme entrapped in a gelatin-based hydrogel without PVA (G/PVA 0%, black), a gelatin-based hydrogel with 1% *w*/*v* of PVA (G/PVA 1%, striped), and a gelatin-based hydrogel with 2% *w*/*v* of PVA (G/PVA 2%, grey). In each case, the concentration of gelatin was 10% *w*/*v*. The value of activity obtained in the first reaction cycle was assumed to be 100%. Mean value of three independent repetitions.

In order to investigate the practical application of the tested materials, we considered whether the produced gelatin-based hydrogels and gelatin/PVA blends could be used as suitable matrices for enzyme immobilization. For this purpose, invertase from *Saccharomyces cerevisiae* was used as a model enzyme and subjected to entrapment in each type of hydrogel carrier. During the study, the immobilization yield and operational stability of the immobilized invertase preparations were determined. It was observed that both the immobilization efficiency (Figure 5) and the operational stability (Figure 6) increase with increasing PVA content in the hydrogel matrices.

According to our previous study [38], the rather high immobilization yield determined for all hydrogel enzyme systems and expressed as a percentage of the retained activity can be reliably explained. Based on the former molecular analysis, we concluded that, apart from entrapment, the invertase could form strong covalent bonds with gelatin chains as a result of cross-linking with transglutaminase (TGase). Moreover, we did not observe any leakage of invertase from each hydrogel carrier. Therefore, the immobilization of the enzyme molecules was considered to be fully permanent. Although in each case the entire amount of protein was trapped in the polymer matrix, the immobilized invertase preparation based on the hydrogel containing 2% PVA (Figure 5, grey) was characterized by the highest catalytic activity.

Due to the increased content of PVA, the flexibility of the hydrogel matrix and its swelling ratio increased as well. This phenomenon provides a better exchange of reactants during the catalytic reaction and complete retention of the enzyme’s molecules in the cross-linked support at the same time. This is particularly noticeable in the results of the operational stability studies (Figure 6). In the case of using the invertase immobilized in the hydrogel-based only on gelatin (Figure 6, black bars), a gradual drop in activity in each subsequent reaction cycle can be observed. This finding could be explained by the clogging of the hydrogel’s pores by reaction products, which hinders the access of the substrate’s molecules to the enzyme’s active center. The application of an enzyme entrapped in a cross-linked gelatin/PVA blend makes it possible to obtain a more stable enzyme preparation with constant activity of over 70% of the initial value (Figure 6, striped and grey bars). However, the highest activity (approximately 90%) was retained by the invertase immobilized in the gelatin hydrogel containing 2% PVA. This is a very promising result that indicates the high application potential of this hydrogel-entrapped biocatalyst in continuous processes or for repeated use in successive reaction cycles.

### 2.2. Computational Studies/Molecular Insight into the Structural Characterization of Gelatin and Gelatin/PVA Hydrogels

The macroscopic properties of the studied material, such as tensile strength, swelling degree, and porosity, and its dependence on operational parameters (temperature) in principle are a function of interactions on the molecular level. A deep understanding of the chemical structure and physical couplings within the studied material provides not only scientific insight but also possible scenarios for modifying its composition to achieve the desired operational response. One of the most popular methods for studying the properties of amorphous materials is molecular dynamics. By treating the molecular system as a collection of point masses coupled by chemical and electrostatic interactions and solving Newton’s equation, one may study the nature of physical interactions within the material and its relation to its measurable properties. Furthermore, computational methods may be and often are, used as virtual replacements for actual experiments, reducing the number of working hours and the funds required to optimize the material’s functionality.

In this work, we intended to study the possibility of predicting the properties of mixed gelatin/PVA systems using the molecular dynamics method.

#### 2.2.1. Models of Gelatin and Gelatin/PVA Blends

**Figure 7:**
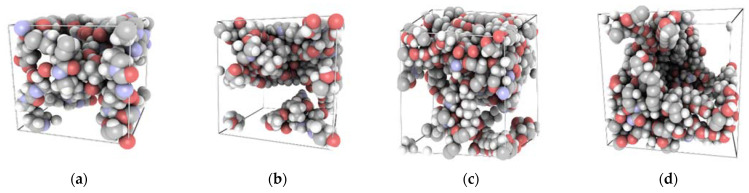
Four gelatin/PVA systems under periodic boundary conditions. The PVA content is: 0% (**a**), 40% (**b**), 62% (**c**), and 70% (**d**).

For the research presented in this article, we used a simplified model of gelatin based on a sequence developed in our previous paper [37]. As a result, the gelatin model was constructed out of three fibers of the following sequence: H_2_N-Pro-Gly-Hyp-Hyp-Gly-Hyp-Pro-Gly-Glu-Gln-Gly-Pro-Ala-Gly-Lys-COOH. Since the gelatin content and degree of PVA polymerization are fixed, the change in composition percentage cannot be continuously modified. The overall process of folding gelatin/PVA blends into an amorphous structure under periodic boundary conditions and using an equilibration protocol is computationally complex, so we narrowed our study down to four different compositions (Figure 7).

#### 2.2.2. Mechanical Properties of Gelatin and Gelatin/PVA Blends

**Figure 8:**
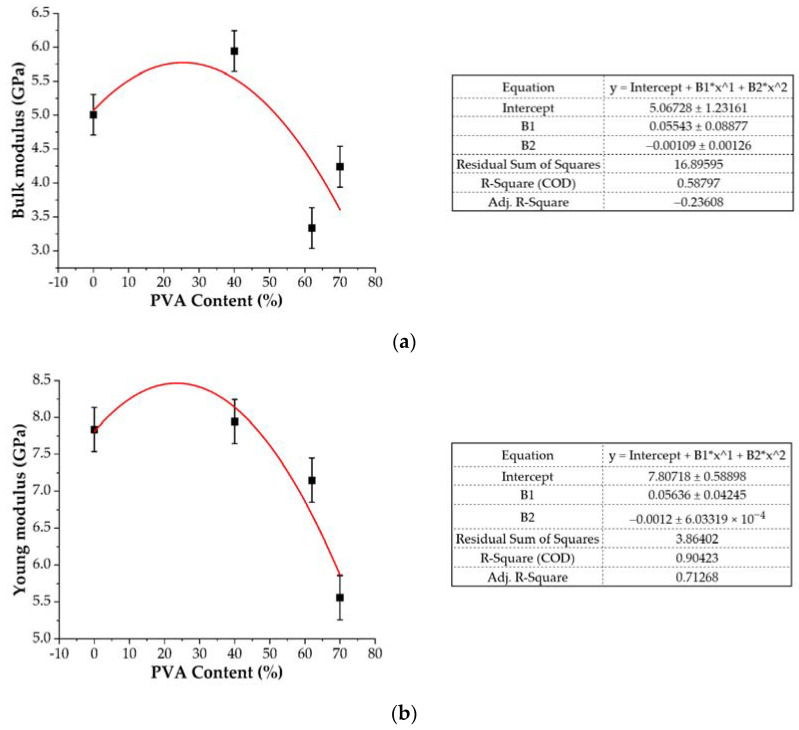
Bulk modulus (**a**) and Young’s modulus (**b**) of equilibrated structures vs. PVA content in gelatin/PVA hydrogels.

As can be observed, the off-diagonal components in the stiffness matrix are non-zero, indicating that the gelatin/PVA blends are not ideal isotropic materials. The diagonal components are an order of magnitude greater than the off-diagonal ones, and the entire matrix is almost symmetric along with the diagonal components. This implies that the critical features of isotropic materials are included in this model and that the static method is applicable for calculating the mechanical properties of the blends. The hardness of a material is closely related to Young’s E modulus; in particular, larger Young’s modulus values suggest a stronger rigidity and hardness. The fracture strength of a material is determined by the Bulk modulus, with a higher value indicating greater fracture resistance [55]. The Young’s modulus of the pure gelatin module using the static method is 7.84 GPa (Figure 8), which is in relative agreement with the experimental value of 2.86 [56] and the result (6.4 GPa) of the static method calculation reported by Zaupa et al. [57]. As one can observe, the Young’s modulus and the Bulk modulus increase up to a PVA concentration of 40% and then gradually decrease (Figure 8) due to the gelatin-gelatin and gelatin-PVA interaction effects. As depicted in Figure 11, the PVA fills the empty spaces in between gelatin chains and in this way the system’s stiffness increases thanks to non-bonded interactions. A further increase in the PVA content introduces, however, a screening effect and a reduction in the overall flexibility of the system due to the reduced number of bonded interactions. As a result, the stiffness of the system is reduced.

In order to explain this phenomenon, we studied the contributions of energy density in the given hydrogel blend. The energy density is defined as:

(1) $$E_{d}=\frac{\sum_{i=1}^{n}\frac{E_{i}}{V_{i}}}{n}\;,$$

where $E_{i}$ is the energy in a given time frame (van der Waals, electrostatic, and bonded energy) and $V_{i}$ is the volume of the simulation box.

**Figure 9:**
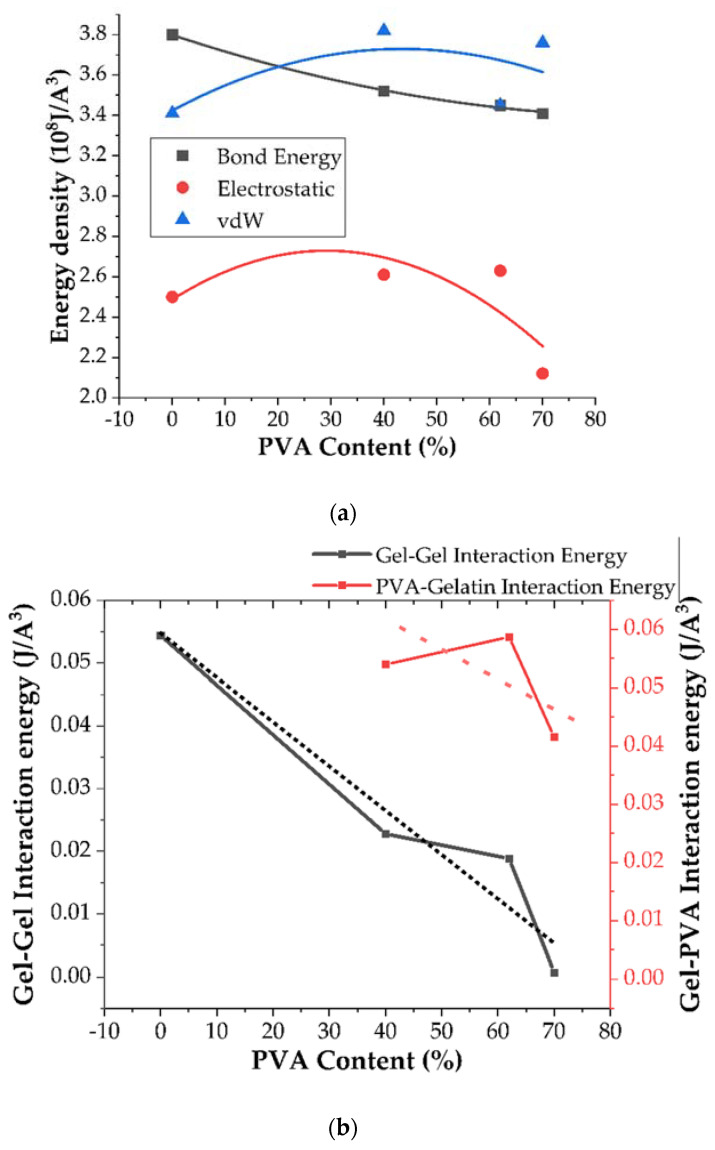
Energy contributions in the gelatin/PVA blends (**a**) and gelatin-gelatin and gelatin-PVA interaction energy (**b**) depending on PVA content.

As one can observe in Figure 9, with increasing PVA content there is a significant decrease in the electrostatic and bonded energy per unit volume, whereas vdW remains constant.

Since the bonded energy contribution may be directly related to the flexibility of the system and the electrostatic and vdW energy contribution with interchain coupling, it is expected that Young’s moduli will decrease. The decrease in the number of mutual interactions between gelatin and PVA fibers results in a decrease in the Bulk modulus and the overall hardness of the material. We associate this phenomenon with the screening properties of the electrostatic coupling between the gelatin fibers and the PVA.

#### 2.2.3. Porosity and Swelling Properties of the Gelatin and Gelatin/PVA Blends

**Figure 10:**
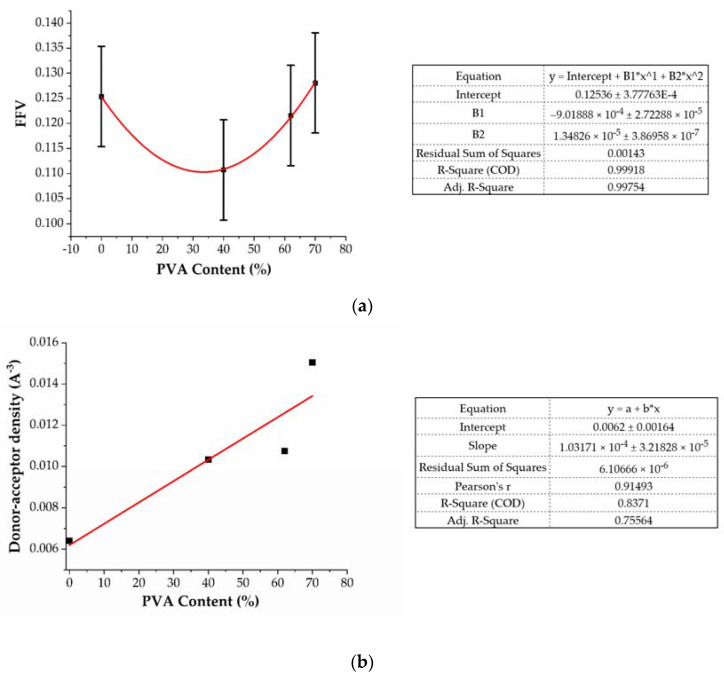
FFV (**a**) and hydrogen bond donor-acceptor density (**b**) depending on the PVA content in the gelatin/PVA blend.

The increase in the swelling degree can be explained if one analyzes the donor-acceptor density and the FFV of the analyzed system (Figure 10). The gelatin’s molar mass is approximately 10,000 g/mol, whereas that of PVA is over 100 times less (86.09 g/mol). Furthermore, each PVA fiber contributes with one hydroxyl group being the hydrogen bond acceptor-donor. Thus, it is expected that the hydrogen bond donor-acceptor density per unit volume should increase because of the increase in the PVA content. Our calculations (Figure 10b) confirm this scenario. Since hydrogen bonding is a major mechanism for water adsorption in hydrogels, and since the elasticity of the network also increases with the PVA content, the increase in the swelling degree is not a surprise. The swelling is also powered by a decrease in the mutual coupling within the gelatin/PVA system.

**Figure 11:**
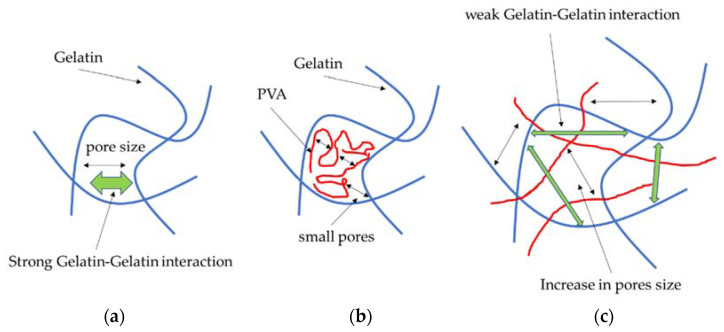
Model of the change in porosity within pure gelatin (**b**) and gelatin/PVA blends (**b**,**c**). The initial pore distribution in the gelatin blend is a result of mutual gelatin-gelatin interactions (green arrows) (**a**). The PVA interacts with gelatin via electrostatic and hydrogen interactions and fills the empty space, reducing the pore size (**b**). The screening effect of PVA increases the pore size distribution (**c**).

The change in porosity of the blend may also be associated with the FFV on the molecular level. The results shown in Figure 10a suggest an initial decrease in the unoccupied volume with the increase in the PVA content. The reason for this is that, due to the smaller molecular mass and greater mobility of the PVA, this polymer fills the empty pores within the gelatin system. The greater affinity of PVA to gelatin results in the penetration of the latter by PVA, so the overall pore distribution shifts towards smaller values (Figure 11).

Thanks to the initial addition of PVA, both the pore size and the FFV within the system, due to the flexibility and strong affinity of PVA to gelatin, decrease. However, with a further increase in the PVA content, the gelatin-gelatin and gelatin-PVA interactions decrease. This may result from a shift of the pore distribution towards larger values.

## 3. Discussion

Due to their favorable bio-application properties, research on hydrogel materials based on gelatin and PVA blends is widely reported in the literature. The articles, in particular, are concerned with using these materials for a variety of medical purposes, including tissue engineering [41,58,59,60,61,62], wound dressings [62,63,64,65,66], drug delivery systems [62,67], implants [68], and vascular grafts [69]. However, other fields of application have also been intensively explored, such as heavy metal removal [70], biodegradable food packaging [40], and enzyme immobilization [71]. It is important to emphasize that, in each case, the properties of gelatin/PVA hydrogels depend not only on the ratio of these components but also on the cross-linking method used and the final form/shape of the received material. These hydrogels can be produced with a variety of cross-linking methods based on using chemical agents (e.g., glutaraldehyde [41,59], sodium trimetaphosphate [69], genipin [60], and microbial transglutaminase [58]) or physical processes, such as irradiation [61,63] and repeated freeze-thaw cycles [58,59,64,65,68]. Furthermore, they can be obtained in the form of thin films [58,63,64,66,69], membranes [67], and three-dimensional porous constructs [41,59,60,61,65,68,69]. Gelatin/PVA hydrogel blends can be based mainly on PVA with a small amount of gelatin [58,59,61,63,64,65,66,67,68,69] as well as quite the opposite, with gelatin as the major component with the addition of a small amount of PVA [41,59,63]. These materials can be also prepared using an equal proportion of both components [60,63]. Finally, considering all possible combinations of component ratios, cross-linking methods, and final shapes, it should be emphasized that it is very challenging to directly compare the properties of the gelatin/PVA blends obtained by different researchers. However, based on selected literature reports, some noticeable convergences/tendencies can be indicated.

In our study, the materials were prepared as cylinder-shaped 3D constructs using a gelatin/PVA ratio of 10:0, 10:1, and 10:2 (% *w*/*v*) and chemical cross-linking with a microbial transglutaminase/borax solution. The analysis of the results obtained from the experimental research led to the conclusion that the increase in the PVA content in gelatin hydrogels contributes to obtaining materials with a visibly greater packaging density, which was expressed by the reduced pore size (Figure 2) and the lower water content in their structure (Table 3). A similar tendency to decrease the pore size distribution with an increase in the PVA ratio has also been noticed by other research groups [41,59,60]. According to the work of Mahnama et al. [41], the proportion of smallest pores (<50 μm) increased from 15% to 70% for a hydrogel based on pure gelatin with the addition of 20% PVA content. In turn, Thangprasert and coworkers observed a decrease in the average pore size from 115 μm (determined for pure gelatin) to 67 μm and 57 μm for materials composed of gelatin:PVA ratio of 30:70 and 0:100, respectively [59]. This direction of change was also described in a research report of Nguyen et al. [60], where the pore size distribution was found to be inversely proportional to the increasing concentration of PVA in the tested materials. They also found that, for the PVA amount of 20% (*w*/*v*), pores with the size of 100–200 µm had the largest proportion in the gelatin/PVA hydrogel structure. For the PVA amount of 50% (*w*/*v*), the average pore size ranged from 5 to 20 µm.

In our research, it was observed that the swelling degree (Table 4) and the water absorption capacity (Table 5) of gelatin/PVA matrices increased with increasing PVA ratio. Similar results were obtained by Mahnama et al. [41] among others. They noticed that for gelatin/PVA blends produced respectively in a 9:1, an 8:2, and a 7:3 ratio for both components, the swelling degree increased as follows: 1370, 1460, and 1750%. We found that this parameter changed for the gelatin/PVA ratios of 10:0, 10:1, and 10:2 as follows: 789.5, 991.2, and 1018%, respectively (determined at 30 °C). In another paper, Nguyen and coworkers showed that changing the PVA concentration from 10 to 30% (*w*/*v*) caused an increase in the swelling capacity of the obtained materials by 50% [60]. This phenomenon could be explained by the presence of numerous hydroxyl groups in the PVA structure that interact very well with water molecules through hydrogen bonds, resulting in a greater ability of the resulting material to soak up various solutions. The greater the proportion of PVA in the hydrogel, the higher the water binding capacity.

In the case of hydrolytic degradation (Table 6) and compressive strength (Table 2), we observed the opposite tendency: both these parameters decreased when the PVA content increased. Similar results were obtained in the work of Nguyen et. al. [60], where an increase in the concentration of PVA from 20 to 50% (*w*/*v*) in the obtained material also caused a significant decrease in these parameters. Nevertheless, in the case of compressive strength, it should be noted that some authors have reported a synergistic increase with an increase in PVA content [59,65]. However, unlike the hydrogels obtained in our work, these results apply primarily to materials in which the major component is PVA.

The functionality studies on gelatin and gelatin/PVA hydrogels for enzyme immobilization performed in the current study indicate the very promising potential of invertase entrapped in a gelatin/PVA hydrogel matrix (Figure 6, grey) as a stable biocatalyst for continuous processes or repeated use in successive reaction cycles of sucrose hydrolysis. This research direction is in line with leading trends in the bioengineering field aimed at developing eco-friendly, clean, and economically justified biotechnologies for the manufacturing processes of various food and pharmaceutical products.

By applying typical macroscopic laboratory research methods, the precise characteristics of the material’s properties can be determined. In this way, accurate analysis of the suitability of the studied hydrogels for specified applications can be carried out. Nevertheless, if one wants to determine why the properties of the resultant materials change with increasing PVA content, the basis for this phenomenon should be sought at the molecular level.

In order to further understand these phenomena, we also employed a molecular modeling technique. Molecular dynamics is a well-established method for investigating and screening polymers and polymer blend systems as indicated in numerous works [72,73,74,75,76,77]. There are, however, only a few works concerning molecular simulations of gelatin [57,78,79]. One possible reason for this is that gelatin is a difficult system to model since it is composed of over 10 amino acids in a pseudo-random order. Hence, the key element is to create a simplified molecular model that reproduces the fundamental properties of interest while conserving the chemical composition of the biopolymer. Such models have been studied in our previous paper [37]. Another problem is the creation of an amorphous structure under periodic boundary conditions, which requires a tacit annealing and equilibration process. Thus, most of the research provides only qualitative results, especially in terms of mechanical properties, and deviations from experimental results are somewhat to be expected.

In this work, we analyzed the problem computationally within two regimes. In the first one, we tried to capture the major tendencies in the physical and chemical properties of gelatin/PVA blends when the content of PVA is gradually increased in the case where the major component of the blend is gelatin (gelatin phase). This analysis was supported by both experimental and theoretical studies. The molecular modeling approach offers, however, the possibility of capturing theoretical scenarios in which the major component in the blend is PVA (PVA phase) and allows us to hypothesize about its properties. Although, in our approach, in the case of molecular modeling, we did not consider the effect of the water concentration, we intended to show that mutual gelatin/PVA interactions correlate with the major physical properties of the blend.

As was observed from the experimental study, the mechanical resistance of the gelatin phase blend increased when the content of PVA was increased. A possible explanation for this effect is the increased affinity of the PVA for gelatin. Since the bonded interactions within a gelatin fiber are rather strong, the pore distribution leaves open space that the PVA can fill. The reduction in the FFV and the experimental results in which the pore distribution in materials with increasing PVA content changed towards smaller values support this claim (Figure 2). As a result of the gelatin-PVA interactions, the PVA fills the pores within the gelatin matrix and forms a scaffold that supports the blend matrix. The sorption capacity of the blend increased due to the increase in the polarity of the system and the hydrogen donor-acceptor density (Figure 10).

It would be interesting to consider the further evolution of the investigated system when PVA is the major component (PVA phase). As one can see in Figure 10, the overall tendencies of the analyzed parameters (FFV, pore distribution, and mechanical strength) may be reversed. The increasing concentration of the PVA due to its polarity may result in a screening effect and an increase in the elasticity of the system. The interaction energy density analysis depicted in Figure 9 suggests that, with increasing PVA content, the bonded interaction density decreases and is supported by a decrease in the gelatin-gelatin and gelatin-PVA coupling effect. As a result, the pore distribution may again shift towards larger values but the mechanical strength of the system will be reduced. This scenario will be verified in upcoming research.

## 4. Materials and Methods

### 4.1. Materials

Porcine skin gelatin (G), polyvinyl alcohol (PVA), sodium tetraborate (borax), and invertase from baker’s yeast (*Saccharomyces cerevisiae*) (EC 3.2.1.26) were supplied by Sigma-Aldrich (Darmstadt, Germany). Microbial transglutaminase (mTGase) Activa^®^WM was kindly donated by Ajinomoto (Tokyo, Japan). Other reagents, all of the analytical grade, were supplied by Avantor Performance Materials (Gliwice, Poland).

### 4.2. Methods

#### 4.2.1. Preparation of Hydrogel Matrices

Gelatin hydrogels differing in PVA content were prepared by the use of the modified procedure described in previous work [80]. Briefly, a weighed portion of gelatin (final concentration: 15% *w*/*v*) and PVA (final concentration: 0.0, 1.5, or 3.0% *w*/*v*) was dissolved in distilled water in a thermostatic reactor at 80 °C. Next, the solution was cooled to 40 °C and incubated at this temperature for approximately 30 min. In the parallel process, a buffer solution of mTGase (final concentration: 3.0% *w*/*v*) was prepared. The cross-linking was begun by mixing an appropriate amount of mTGase with gelatin or gelatin/PVA solution at a volume ratio of 1:2. Afterward, the obtained blend was immediately cooled to 4 °C and kept under these conditions for 24 h. Finally, in order to cross-link PVA chains into a three-dimensional polymer network, the obtained hydrogel particles were placed in 4% (*w*/*v*) borax solution for 1 h. Then, the obtained product was rinsed twice with distilled water and dried at 60 °C until a constant weight was reached.

In each case, the final concentration of gelatin was constant (10% *w*/*v*), but the materials differed in the content of PVA (0, 1, or 2% *w*/*v*).

#### 4.2.2. Determination of Pore Distribution in Hydrogel Materials

In order to determine the pore distribution in the tested hydrogels, the samples of dried materials were imaged using a SEM/Ga-FIB FEI Helios NanoLab™ 600i microscope (Thermo Fisher Scientific, Waltham, MA, USA). However, it should be pointed out that the dry hydrogel samples were non-conductive. Therefore, initially, it was necessary to apply a thin conductive carbon layer to them using a Quorum Technologies Q150T E vacuum coater (Quorum Technologies, Lewes, UK). Then, each sample was placed in a high-resolution scanning electron microscope (SEM) coupled to an ion microscope that uses the focused ion beam (FIB) technology. However, as a side effect of the drying process applied to the hydrogels, the pores were not visible on the surface of the samples. In order to remove the surface layer, the material was first exposed to an ion beam for 30 s. In this way, the pore structure was revealed again. Then, an image of the exposed area was recorded, and the pore size analysis was performed on the captured images.

A detailed analysis of the pore distribution was performed for each sample on 30 such images using ImageJ software (NIH, Bethesda, MD, USA). In each case, the analyzed area was 20 × 20 microns. In order to perform the analysis, each of the resulting images was binarized (the pores were marked in black and the area without pores in white). It should be noted that some images required binarization several times because there were large pores with smaller pores inside them. In this case, at least two binarized images were created (one for large pores and one for small pores). This image processing procedure enabled the precise identification of the pore size, allowed us to count the number of pores, and, finally, enabled us to produce detailed histograms of the pore distribution for each hydrogel material.

#### 4.2.3. Determination of the Mechanical Properties of Hydrogels

**Figure 12:**
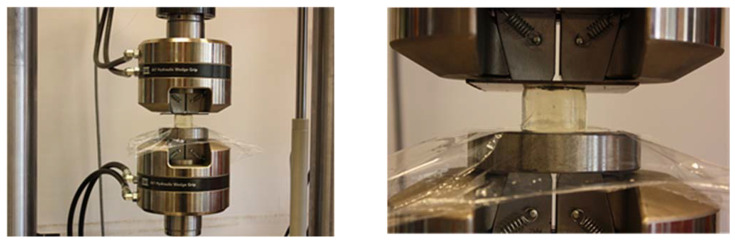
MTS Bionix mechanical testing system equipped with a 1-kN load cell.

The basic method used to determine the mechanical properties of a material is tensile or compression testing. The hydrogels examined in this paper could not be tested by tensile testing due to their consistency. Therefore, the mechanical properties were determined in a compression test at ambient temperature. Cylindrical samples (Ø32 × 30 mm) in the as-prepared state were tested. Figure 12 shows the sample configuration and the test setup. The test was performed with the use of an MTS Bionix mechanical testing system (MTS Systems Corporation, Eden Prairie, MN, USA) equipped with a 1-kN load cell (class 1). The samples were compressed between two flat plates at a fixed displacement rate of 30 mm/min until failure while the load and displacement were recorded. The compressive stress and strain were calculated as load/area and displacement/initial height, respectively.

#### 4.2.4. Determination of the Water Content in the Hydrogel Structures

Initially, each type of hydrogel (cylindrical shape, Ø32 × 30 mm) was weighed. Next, the material samples were dried at 60 °C until a constant mass was reached. Then, the obtained xerogels were weighed again. The amount of water in each hydrogel structure was calculated as the difference between the initial weight of the hydrated material sample and the weight of the same sample after drying:

(2) $$Water\;content\;\left(\%\right)=\frac{W_{INITIAL,WET}-W_{INITIAL,DRY}}{W_{INITIAL,WET}}\times 100\;,$$

where *W_INITIAL,WET_* and *W_INITIAL,DRY_* are the weights of the initial wet sample and the dry hydrogel, respectively.

Experiments on each type of hydrogel tested were run in triplicate. The results are presented as the average values with a standard deviation of ±2.1–5.3%.

#### 4.2.5. Determination of Hydrogel Sorption Properties

For each type of hydrogel, a swelling experiment was performed by placing one dried particle in 50 mL of distilled water in a thermostatic bath (30, 40, or 50 °C) for 24 h. Then, the swollen hydrogel was removed from the water, quickly and carefully drained with a paper towel, and weighed. As a result, the swelling degree (SD) was calculated using the following equation [80]:

(3) $$SD\;\left(\%\right)=\frac{W_{SWOLLEN,WET}-W_{INITIAL,DRY}}{W_{INITIAL,DRY}}\times 100\;,$$

where *W_INITIAL,DRY_* and *W_SWOLLEN,WET_* are the weights of the initial dry sample (xerogel) and the wet swollen hydrogel, respectively.

Next, the swollen hydrogel was dried again at 60 °C until a constant mass was reached, weighed again, and then the water absorption capacity (WA) and the rate of hydrolytic degradation (HD) [80] were determined. The following equations were used:

(4) $$WA\;\left(\%\right)=\frac{W_{SWOLLEN,WET}-W_{SWOLLEN,DRY}}{W_{SWOLLEN,DRY}}\times 100\;,$$

(5) $$HD\;\left(\%\right)=\frac{W_{INITIAL,DRY}-W_{SWOLLEN,DRY}}{W_{INITIAL,DRY}}\times 100\;,$$

where *W_INITIAL,DRY_*, *W_SWOLLEN,WET_,* and *W_SWOLLEN,DRY_* are the weights of the initial dry hydrogel, the wet swollen hydrogel, and the dry hydrogel after swelling and re-drying, respectively.

For all materials tested, the swelling experiments were run in triplicate. In this paper, the results are presented as the average values with a standard deviation of ±2.1–5.3%.

#### 4.2.6. Entrapment of Invertase from *Saccharomyces Cerevisiae* in Hydrogel Matrices

Immobilization of invertase in gelatin and gelatin/PVA-based hydrogels was performed analogously to the method applied in our previous study [38].

Briefly, a weighed portion of gelatin (15% *w*/*v*) and PVA (0.0, 1.5, 3.0% *w*/*v*) was dissolved in 0.05 M acetate buffer at pH 4.5 in a thermostatic reactor at 80 °C. Next, the solution was cooled to 40 °C and incubated at this temperature for approximately 30 min. In parallel, a buffer solution of invertase with a given concentration was prepared. Then, after the total dissolution of the enzyme, a weighted portion of cross-linking agent (microbial transglutaminase, mTGase) was added to a concentration of 3% *w*/*v*. The crosslinking was begun by mixing an appropriate amount of mTGase with gelatin or gelatin/PVA solution at a volume ratio of 1:2. Afterward, the obtained blend was immediately cooled to 4 °C and kept under these conditions for 24 h. Finally, in order to cross-link PVA chains into a three-dimensional polymer network, the obtained hydrogel particles were placed in a 2% (*w*/*v*) borax solution for 1 h then rinsed twice with 0.05 M acetate buffer at pH 4.5. After this time, the (cylinder-shaped) hydrogel particles were drained, washed twice with 0.05 M acetate buffer at pH 4.5, and their geometric dimensions were measured (d = 1.2 mm; h = 0.8 mm). It was assumed that the total amount of used invertase was immobilized in the hydrogel cross-linked network. The experiment was run in triplicate.

Immobilization yield was determined as the activity of the bound enzyme divided by the activity of the native form of the enzyme and is expressed as a percentage.

#### 4.2.7. Determination of Protein Concentration

The protein concentration was determined using Lowry’s method [81] with bovine serum albumin applied as a protein standard. This procedure consists of two steps: a biuret reaction and the reduction of Folin-Ciocalteu reagent to the corresponding oxides with Cu^2+^ and tyrosine and tryptophan residues. In this method, the intensity of the blue color of the product measured spectrophotometrically at 750 nm is directly proportional to the protein concentration in the analyzed sample.

The test was performed as follows: 0.5 mL of Lowry reagent was added to a 0.5 mL solution of the analyzed sample, mixed, and incubated at room temperature for 20 min. Next, 0.25 mL of Folin-Ciocalteu reagent was added, immediately mixed, and incubated for another 30 min. Then, the absorbance at 750 nm was measured. The protein concentration was determined using the standard curve (C_PROTEIN_ (µg/mL) = 207.9.A750) obtained for different concentrations of bovine serum albumin in the range 10–200 µg/mL.

#### 4.2.8. Determination of Glucose Concentration

Glucose concentration was determined using a commercially available analytical test (Lublin, Biomaxima, Poland). This detection method is based on the conversion of glucose to the colored product formed as a result of two consecutive enzymatic reactions: oxidation of glucose to gluconic acid and hydrogen peroxide and subsequent reaction of the hydrogen peroxide with hydroxybenzoic acid (HBA) and 4-aminoantipyrine (AAP). A red dye (quinoneimine) is obtained as the final product of these reactions. The intensity of the produced material’s color is measured spectrophotometrically at 500 nm. It is known that the intensity is directly proportional to the glucose concentration in the sample.

The test was performed as follows. First, 10 μL of the sample withdrawn from the reaction mixture was added to 1 mL of analytical reagent and incubated for 5 min at 37 °C. Then, the absorbance at 500 nm was measured. Finally, the glucose concentration was determined using the glucose standard (C_STANDARD_ = 1.0 g L^−1^) as the reference.

#### 4.2.9. Determination of the Catalytic Activity of Native and Immobilized Invertase

The catalytic activity of invertase was determined by monitoring the hydrolysis of sucrose to glucose and fructose over time. In all experiments, the sucrose solution at a final concentration of 50 g L^−1^ prepared in 0.05 M acetate buffer (pH 4.5) was used as a substrate. The activity determination method was based on measuring the concentration of the glucose that formed as a reaction product. One unit of enzyme activity (1 U) was defined as 1 µg of glucose formed within 1 min of the reaction of sucrose hydrolysis catalyzed by invertase.

The reaction with the native enzyme was carried out in a sealed test tube. A total of 5 mL of sucrose solution was preincubated for 10 min in a thermostatic water bath at 50 °C. After this time, the reaction was started by adding 0.1 mL of invertase to the substrate. During the 5-min process, the samples were withdrawn every 0.5 min for the determination of glucose content.

The reaction using immobilized invertase was carried out in a thermostatic reactor with stirring (250 rpm) at 50 °C. A total of 45 mL of 50 g L^−1^ sucrose solution was pre-incubated for 10 min, and then the reaction was started by adding one bead of the hydrogel with immobilized enzyme (V_BEAD_ = 0.9 mL). During the 10-min process, samples were taken every 1 min for the determination of glucose concentration.

#### 4.2.10. Determination of the Operational Stability of Invertase Immobilized in Hydrogel Matrices

The operational stability of immobilized invertase was determined in 10 consecutive batch processes of sucrose hydrolysis (reaction time: 30 min). For that purpose, all the enzyme-hydrogel preparations were examined. Ten subsequent reactions were performed for each sample of entrapped invertase in triplicate. Each reaction was carried out in a thermostatic reactor with stirring (250 rpm) at 50 °C. A total of 45 mL of 50 g L^−1^ sucrose solution was preincubated for 10 min, and then the reaction was started by adding one bead of the hydrogel with immobilized enzyme (V_BEAD_ = 0.9 mL). During the 30-min process, samples were taken every 1 min for the determination of glucose concentration. After the end of one reaction, the hydrogel-bound enzyme was washed twice with 0.05 M acetate buffer at pH 4.5 and the next batch process was started with the same hydrogel-bound invertase particle. The reaction rate received in the first run was set as 100%.

#### 4.2.11. Preparation of Molecular Models of Hydrogel Structures

**Figure 13:**
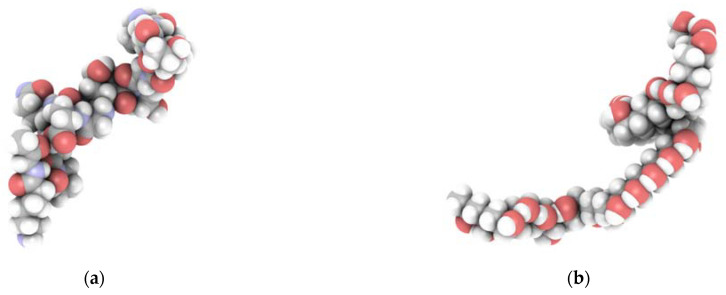
The gelatin (**a**) and PVA (**b**) molecular structures used in the simulation. An appropriate blend of both results in a simulation system under periodic boundary conditions.

To study the molecular properties of gelatin/PVA systems (Figure 13), we used the Polymatic tool (Department of Materials Science and Engineering, The Pennsylvania State University, State College, PA, USA) [82]. Using PCFF forcefield, we created 12 gelatin/PVA systems with four different concentrations of PVA (0%, 40%, 62%, and 70%) under periodic boundary conditions. The protocol involves a Monte Carlo search of the torsion angles between single PVA units and subsequent minimization in a given force field. This procedure ensures that the resultant structure is at an energetic minimum and thus provides a reliable initial system for further dynamical calculations. Nonetheless, the models were then equilibrated using molecular dynamics as implemented in the LAMMPS simulation package. The equations of motion were integrated using the Velocity Verlet algorithm using a 1 fs time step. The studied system is highly amorphous and has a large number of degrees of freedom. Therefore, it requires an equilibration protocol that prevents the system from occupying a high-energy metastable state. Thus, we derived an equilibration protocol that includes heating up and careful cooling down steps. First, the system was heated up to 600 K in the NPT canonical ensemble using a Nose-Hoover thermostat and an Andersen barostat (1 Pa). Then, the system was equilibrated for 300 ps and after that gradually cooled down by 50 K in each step. In each run, the temperature and pressure were equilibrated for 50 ps until the next step began. Upon reaching the desired final temperature, i.e., 298 K, the system was equilibrated for another 500 ps in the NPT ensemble and then for another 500 ps in the NVT ensemble. This procedure leads to a final density of 1.26 g/cm^3^, which is in good agreement with the experimental value of 1.35 g/cm^3^ [83].

#### 4.2.12. Computational Determination of Functional Parameters of Hydrogels

In this work, two fundamental properties were studied: Young’s modulus and swelling degree.

In this research, the static method was used to estimate the elastic constants of the gelatin/PVA network. Stress-strain behavior in linear elastic materials can be described by the generalized Hook’s law, which is written as follows.

(6) $$\sigma_{ij}=C_{ij}\epsilon_{j}\;,$$

where $\sigma_{ij}$ is the stress vector, $\epsilon_{j}$ is the strain vector, and $C_{ij}$ is a six-dimensional stiffness matrix.

As polymer blends can be assumed to be isotropic materials, Young’s modulus of interest can be derived from the stiffness matrix as follows.

(7) $$E=\frac{\mu \left(3\lambda +2\mu \right)}{\lambda +\mu }\;,$$

The Lame coefficients μ and λ  can be calculated from the elastic coefficients according to the statistical mechanics of elasticity [84].

(8) $$\lambda =\frac{1}{6}\left(C_{12}+C_{13}+C_{21}+C_{23}+C_{31}+C_{32}\right)\;,$$

(9) $$\mu =\frac{1}{3}\left(C_{44}+C_{55}+C_{66}\right),$$

The static mechanical properties of gelatin/PVA systems were obtained by analyzing the equilibrium models at the temperature of 298 K.

## 5. Conclusions

Biodegradable hydrogels are sustainable materials with many valuable properties that enable their utilization in versatile applications. Due to their biocompatibility, semi-permeability, high water content, and ability to create three-dimensional systems, these hydrogels stand out from other polymeric materials and are significantly similar to natural tissues and other multilayer biological systems. Therefore, they can be used with great success, especially in the life science sector as carriers of bioactive compounds (e.g., drugs, enzymes, antibodies, stem cells), controlled release systems, biosensors, functional dressings, implants, and tissue scaffolds. An invaluable advantage of biodegradable hydrogel matrices is the ability to fine-tune their properties for a specific application. This is done by creating blends of two or more hydrogels that vary in their physicochemical features.

In our research, we prepared just such a type of hydrogel material based on well-known components with desirable biofunctional properties—gelatin and PVA. By determining the dependence of changes in particular properties (e.g., pore size distribution, swelling capacity, and mechanical strength) on increases in PVA content, it became possible to effectively tailor the functionality of the materials for the targeted application, namely a carrier of a bioactive compound with a strictly defined molecular size. In our work, we strived to demonstrate the great importance of the physicochemical characteristics to the efficient use of gelatin/PVA hydrogel blends as a matrix for the immobilization of invertase from baker’s yeast (*Saccharomyces cerevisiae*). From the experimental part of our study, we noticed that due to the increased content of PVA, the flexibility of the hydrogel matrix and its swelling degree also increased. This phenomenon provides a better exchange of reactants during the catalytic reaction. At the same time, the complete retention of the invertase molecules in the cross-linked support is ensured by the formation of additional covalent bonds between the enzyme and the pendant groups of the gelatin as a result of interactions with mTGase.

Typically, the characteristics of obtained materials are determined in direct experimental research. This approach is consistent with good practices and commonly applied; however, it requires a considerable amount of work and expenditure of time and funds. Therefore, new tools are being developed to enable the preliminary design of mixed hydrogel materials with specific properties prior to factual experiments. Thus, the development of a reliable numerical method may save a significant amount of time and reduce the financial outlays required to carry out the research in the laboratory.

The molecular modeling analysis proposed in our work does not provide quantitative results (in terms of a direct relation between the PVA content and the analyzed parameters). It does, however, provide qualitative and very important information, namely the tendencies of the macroscopic parameters observed with an increase in the PVA content and insight into the chemical nature of these dependencies. The difference between the experimental values and the theoretical values can naturally be explained by our not taking into account the effect of the solvent. The presence of water molecules in the system, as one can see from the experimental and theoretical parts of the presented study, has a modulating, rather than a governing, effect on the physical behavior of the system. The inclusion of water in the molecular analysis in the case of the hydrogel would be rather difficult due to the rather small concentration of the polymer blend in the water. This raises difficulties due to the size of the system and the equilibration protocol.

To date, molecular modeling has been successfully and routinely applied in drug design as well as composite engineering where materials with different properties are combined together to provide new functionality. In our opinion, the molecular modeling approach has the power to provide invaluable insight into chemical interactions and their effect on the physical properties of a diverse range of polymeric materials, including hydrogels and their blends. Hence, it may be used to screen for and explain the properties of biomaterials that play a key role in the overall economy. Thus, the experimental procedure presented in this paper may be applied as a valuable tool to tune the properties of these materials and identify new paths of development.

## Figures and Tables

**Table 1:** Tangent modulus of gelatin and gelatin/PVA-based hydrogels determined at a given strain value for one representative sample of each type of material.

Hydrogel Type	Tangent Modulus at Specified Strain (MPa)					
	10%	20%	30%	40%	50%	60%
G/PVA 0%	0.044	0.107	0.147	0.338	0.732	2.314
G/PVA 1%	0.055	0.096	0.191	0.368	0.924	- *
G/PVA 2%	0.071	0.113	0.211	0.437	1.104	- *

* could not be designated due to material failure.

**Table 2:** Compressive strength for gelatin and gelatin/PVA-based hydrogels.

Hydrogel Type	Failure Strength (MPa)						Standard Deviation
	1	2	3	4	5	Average	
G/PVA 0%	0.629	0.763	0.604	0.594	0.440	0.606	0.115
G/PVA 1%	0.250	0.254	0.265	0.272	0.269	0.262	0.009
G/PVA 2%	0.198	0.175	0.187	0.198	0.191	0.190	0.009

**Table 3:** Water content in gelatin and gelatin/PVA hydrogels.

Hydrogel Type	Hydrated Hydrogel (g)	Dried Hydrogel (g)	Water Content in the Hydrogel (%)
G/PVA 0%	15.022 ± 0.466	1.609 ± 0.053	89.29
G/PVA 1%	14.807 ± 0.434	1.703 ± 0.046	88.50
G/PVA 2%	14.006 ± 0.364	1.677 ± 0.051	88.02

**Table 4:** The swelling degree (SD) of gelatin and gelatin/PVA hydrogels was determined after 24 h of incubation at different temperatures.

Hydrogel Type	Swelling Degree (%)		
	30 °C	40 °C	50 °C
G/PVA 0%	481.5 ± 11.56	514.0 ± 16.45	528.5 ± 17.44
G/PVA 1%	547.7 ± 16.98	588.3 ± 25.30	637.6 ± 18.49
G/PVA 2%	626.2 ± 18.16	642.0 ± 32.74	661.0 ± 25.78

**Table 5:** The water absorption capacity (WA) of gelatin and gelatin/PVA hydrogels was determined after 24 h of incubation at different temperatures.

Hydrogel Type	Water Absorption Capacity (%)		
	30 °C	40 °C	50 °C
G/PVA 0%	789.5 ± 16.58	840.5 ± 31.94	853.5 ± 34.99
G/PVA 1%	991.2 ± 35.68	1046 ± 25.10	1267 ± 41.81
G/PVA 2%	1018 ± 48.86	1093 ± 57.93	1348 ± 35.05

**Table 6:** The rate of hydrolytic degradation (HD) of gelatin and gelatin/PVA hydrogels was determined after 24 h of incubation at different temperatures.

Hydrogel Type	Hydrolytic Degradation (%)		
	30 °C	40 °C	50 °C
G/PVA 0%	39.99 ± 0.9197	41.68 ± 1.292	42.06 ± 1.052
G/PVA 1%	34.62 ± 1.004	37.81 ± 1.059	38.81 ± 1.125
G/PVA 2%	31.44 ± 1.132	32.71 ± 0.850	33.36 ± 0.767

## Data Availability

Not applicable.

## References

[1] Slaughter B.V., Khurshid S.S., Fisher O.Z., Khademhosseini A., Peppas N.A. (2009). Hydrogels in regenerative medicine. Adv. Mater..

[2] Aswathy S.H., Narendrakumar U., Manjubala I. (2020). Commercial hydrogels for biomedical applications. Heliyon.

[3] Mallick S.P., Suman D.K., Singh B.N., Srivastava P., Siddiqui N., Yella V.R., Madhual A., Vemuri P.K. (2020). Strategies toward development of biodegradable hydrogels for biomedical applications. Polym. Technol. Mater..

[4] Elkhoury K., Morsink M., Sanchez-Gonzalez L., Kahn C., Tamayol A., Arab-Tehrany E. (2021). Biofabrication of natural hydrogels for cardiac, neural, and bone Tissue engineering Applications. Bioact. Mater..

[5] Kharkar P.M., Kiick K.L., Kloxin A.M. (2013). Designing degradable hydrogels for orthogonal control of cell microenvironments. Chem. Soc. Rev..

[6] Tan H., Marra K.G. (2010). Injectable, biodegradable hydrogels for tissue engineering applications. Materials.

[7] Huang H., Qi X., Chen Y., Wu Z. (2019). Thermo-sensitive hydrogels for delivering biotherapeutic molecules: A review. Saudi Pharm. J..

[8] Kamath K.R., Park K. (1993). Biodegradable hydrogels in drug delivery. Adv. Drug Deliv. Rev..

[9] Peers S., Montembault A., Ladavière C. (2020). Chitosan hydrogels for sustained drug delivery. J. Control. Release.

[10] Ahmed I., Elsherif M., Omar A., Saadi W., Ali M., Alqattan B., Salih A., Saleh H., Al Qutayri M., Park S. (2020). Usage of Hydrogels for Brian Imaging and Diagnostics. Glob. J. Eng. Sci..

[11] Wiraja C., Ning X., Cui M., Xu C. (2020). Hydrogel-Based Technologies for the Diagnosis of Skin Pathology. Technologies.

[12] Culver H.R., Clegg J.R., Peppas N.A. (2017). Analyte-Responsive Hydrogels: Intelligent Materials for Biosensing and Drug Delivery. Acc. Chem. Res..

[13] Klein M., Poverenov E. (2020). Natural biopolymer-based hydrogels for use in food and agriculture. J. Sci. Food Agric..

[14] Khalesi H., Lu W., Nishinari K., Fang Y. (2020). New insights into food hydrogels with reinforced mechanical properties: A review on innovative strategies. Adv. Colloid Interface Sci..

[15] Yang J., Shen M., Luo Y., Wu T., Chen X., Wang Y., Xie J. (2021). Advanced applications of chitosan-based hydrogels: From biosensors to intelligent food packaging system. Trends Food Sci. Technol..

[16] Mitura S., Sionkowska A., Jaiswal A. (2020). Biopolymers for hydrogels in cosmetics: Review. J. Mater. Sci. Mater. Med..

[17] Rizwan M., Rubina Gilani S., Iqbal Durani A., Naseem S. (2021). Materials diversity of hydrogel: Synthesis, polymerization process and soil conditioning properties in agricultural field. J. Adv. Res..

[18] Mikula K., Izydorczyk G., Skrzypczak D., Mironiuk M., Moustakas K., Witek-Krowiak A., Chojnacka K. (2020). Controlled release micronutrient fertilizers for precision agriculture—A review. Sci. Total Environ..

[19] Kalossaka L.M., Sena G., Barter L.M.C., Myant C. (2021). Review: 3D printing hydrogels for the fabrication of soilless cultivation substrates. Appl. Mater. Today.

[20] Singh N., Agarwal S., Jain A., Khan S. (2021). 3-Dimensional cross linked hydrophilic polymeric network “hydrogels”: An agriculture boom. Agric. Water Manag..

[21] Gyles D.A., Castro L.D., Silva J.O.C., Ribeiro-Costa R.M. (2017). A review of the designs and prominent biomedical advances of natural and synthetic hydrogel formulations. Eur. Polym. J..

[22] Liu X., Liu J., Lin S., Zhao X. (2020). Hydrogel machines. Mater. Today.

[23] Mahinroosta M., Jomeh Farsangi Z., Allahverdi A., Shakoori Z. (2018). Hydrogels as intelligent materials: A brief review of synthesis, properties and applications. Mater. Today Chem..

[24] Raghuwanshi V.S., Garnier G. (2019). Characterisation of hydrogels: Linking the nano to the microscale. Adv. Colloid Interface Sci..

[25] Bilal M., Iqbal H.M.N. (2019). Naturally-derived biopolymers: Potential platforms for enzyme immobilization. Int. J. Biol. Macromol..

[26] Dreiss C.A. (2020). Hydrogel design strategies for drug delivery. Curr. Opin. Colloid Interface Sci..

[27] Abaee A., Mohammadian M., Jafari S.M. (2017). Whey and soy protein-based hydrogels and nano-hydrogels as bioactive delivery systems. Trends Food Sci. Technol..

[28] Amiri M., Khazaeli P., Salehabadi A., Salavati-Niasari M. (2021). Hydrogel beads-based nanocomposites in novel drug delivery platforms: Recent trends and developments. Adv. Colloid Interface Sci..

[29] Dhand A.P., Galarraga J.H., Burdick J.A. (2021). Enhancing Biopolymer Hydrogel Functionality through Interpenetrating Networks. Trends Biotechnol..

[30] Kamoun E.A., Chen X., Mohy Eldin M.S., Kenawy E.R.S. (2015). Crosslinked poly(vinyl alcohol) hydrogels for wound dressing applications: A review of remarkably blended polymers. Arab. J. Chem..

[31] Qi X., Su T., Zhang M., Tong X., Pan W., Zeng Q., Shen J. (2020). Sustainable, flexible and biocompatible hydrogels derived from microbial polysaccharides with tailorable structures for tissue engineering. Carbohydr. Polym..

[32] Sharma S., Tiwari S. (2020). A review on biomacromolecular hydrogel classification and its applications. Int. J. Biol. Macromol..

[33] Ullah F., Othman M.B.H., Javed F., Ahmad Z., Akil H.M. (2015). Classification, processing and application of hydrogels: A review. Mater. Sci. Eng. C.

[34] Srivastava N., Choudhury A.R. (2021). Recent advances in composite hydrogels prepared solely from polysaccharides. Colloids Surf. B Biointerfaces.

[35] Catoira M.C., Fusaro L., Di Francesco D., Ramella M., Boccafoschi F. (2019). Overview of natural hydrogels for regenerative medicine applications. J. Mater. Sci. Mater. Med..

[36] Ahmed E.M. (2015). Hydrogel: Preparation, characterization, and applications: A review. J. Adv. Res..

[37] Radosinski L., Labus K., Zemojtel P., Wojciechowski J.W. (2019). Development and validation of a virtual gelatin model using molecular modeling computational tools. Molecules.

[38] Labus K., Wolanin K., Radosiński Ł. (2020). Comparative Study on Enzyme Immobilization Using Natural Hydrogel Matrices—Experimental Studies Supported by Molecular Models Analysis. Catalysts.

[39] Manoochehri H., Hosseini N.F., Saidijam M., Taheri M., Rezaee H., Nouri F. (2020). A review on invertase: Its potentials and applications. Biocatal. Agric. Biotechnol..

[40] Abdullah Z.W., Dong Y., Davies I.J., Barbhuiya S. (2017). PVA, PVA Blends, and Their Nanocomposites for Biodegradable Packaging Application. Polym. Plast. Technol. Eng..

[41] Mahnama H., Dadbin S., Frounchi M., Rajabi S. (2017). Preparation of biodegradable gelatin/PVA porous scaffolds for skin regeneration. Artif. Cells, Nanomed. Biotechnol..

[42] Djagny K.B., Wang Z., Xu S. (2001). Gelatin: A valuable protein for food and pharmaceutical industries: Review. Crit. Rev. Food Sci. Nutr..

[43] Journal T.E. (2006). Opinion of the Scientific Panel on food additives, flavourings, processing aids and materials in contact with food (AFC) related to the use of polyvinyl alcohol as a coating agent for food supplements. EFSA J..

[44] Jain N., Singh V.K., Chauhan S. (2017). A review on mechanical and water absorption properties of polyvinyl alcohol based composites/films. J. Mech. Behav. Mater..

[45] Nagarkar R., Patel J. (2019). Polyvinyl Alcohol: A Comprehensive Study. Acta Sci. Pharm. Sci..

[46] Hou Y., Chen C., Liu K., Tu Y., Zhang L., Li Y. (2015). Preparation of PVA hydrogel with high-transparence and investigations of its transparent mechanism. RSC Adv..

[47] Han J., Lei T., Wu Q. (2014). High-water-content mouldable polyvinyl alcohol-borax hydrogels reinforced by well-dispersed cellulose nanoparticles: Dynamic rheological properties and hydrogel formation mechanism. Carbohydr. Polym..

[48] Schrieber R., Gareis H. (2007). Gelatine Handbook: Theory and Industrial Practice.

[49] Alipal J., Mohd Pu’ad N.A.S., Lee T.C., Nayan N.H.M., Sahari N., Basri H., Idris M.I., Abdullah H.Z. (2019). A review of gelatin: Properties, sources, process, applications, and commercialisation. Mater. Today Proc..

[50] Liang H.C., Chang W.H., Liang H.F., Lee M.H., Sung H.W. (2004). Crosslinking structures of gelatin hydrogels crosslinked with genipin or a water-soluble carbodiimide. J. Appl. Polym. Sci..

[51] Kirchmajer D.M., Watson C.A., Ranson M., Panhuis M. (2013). In Het Gelapin, a degradable genipin cross-linked gelatin hydrogel. RSC Adv..

[52] Yang G., Xiao Z., Long H., Ma K., Zhang J., Ren X., Zhang J. (2018). Assessment of the characteristics and biocompatibility of gelatin sponge scaffolds prepared by various crosslinking methods. Sci. Rep..

[53] Haiyan L., Kunlong M., Zhenghua X., Xiaomei R., Gang Y. (2017). Preparation and characteristics of gelatin sponges crosslinked by microbial transglutaminase. PeerJ.

[54] Yung C.W., Wu L.Q., Ullman J.A., Payne G.F., Bentley W.E., Barbari T.A. (2007). Transglutaminase crosslinked gelatin as a tissue engineering scaffold. J. Biomed. Mater. Res. A..

[55] Wei Q., Cai X., Guo Y., Wang G., Guo Y., Lei M., Song Y., Yingfeng Z., Wang Y. (2019). Atomic-scale and experimental investigation on the micro-structures and mechanical properties of PLA blending with CMC for additive manufacturing. Mater. Des..

[56] Asma C., Meriem E., Mahmoud B., Djaafer B. (2014). Physicochemical characterization of gelatin-cmc composite edibles films from polyion-complex hydrogels. J. Chil. Chem. Soc..

[57] Zaupa A., Neffe A.T., Pierce B.F., Lendlein A., Hofmann D. (2011). A molecular dynamic analysis of gelatin as an amorphous material: Prediction of mechanical properties of gelatin systems. Int. J. Artif. Organs.

[58] Hago E.E., Li X. (2013). Interpenetrating polymer network hydrogels based on gelatin and PVA by biocompatible approaches: Synthesis and characterization. Adv. Mater. Sci. Eng..

[59] Thangprasert A., Tansakul C., Thuaksubun N., Meesane J. (2019). Mimicked hybrid hydrogel based on gelatin/PVA for tissue engineering in subchondral bone interface for osteoarthritis surgery. Mater. Des..

[60] Nguyen T.-H., Ventura R., Min Y.-K., Lee B.-T. (2016). Genipin Cross-Linked Polyvinyl Alcohol-Gelatin Hydrogel for Bone Regeneration. J. Biomed. Sci. Eng..

[61] You S.J., Ahn W.S., Jang H.S., Kang M.I., Chun H.J., Lim Y.M., Nho Y.C. (2007). Preparation and characterization of gelatin-poly(vinyl alcohol) hydrogels for three-dimensional cell culture. J. Ind. Eng. Chem..

[62] Rodríguez-Rodríguez R., Espinosa-Andrews H., Velasquillo-Martínez C., García-Carvajal Z.Y. (2020). Composite hydrogels based on gelatin, chitosan and polyvinyl alcohol to biomedical applications: A review. Int. J. Polym. Mater. Polym. Biomater..

[63] Hubner P., Marcilio N.R., Tessaro I.C. (2021). Gelatin/poly(vinyl alcohol) based hydrogel film—A potential biomaterial for wound dressing: Experimental design and optimization followed by rotatable central composite design. J. Biomater. Appl..

[64] Ren T., Gan J., Zhou L., Chen H. (2020). Physically crosslinked hydrogels based on poly (vinyl alcohol) and fish gelatin for wound dressing application: Fabrication and characterization. Polymers.

[65] Kim S., Lim H., Kim S., Lee D.Y. (2020). Effect of PVA Concentration on Strength and Cell Growth Behavior of PVA/gelatin Hydrogels for Wound Dressing. J. Biomed. Eng. Res..

[66] Imtiaz N., Niazi M.B.K., Fasim F., Khan B.A., Bano S.A., Shah G.M., Badshah M., Menaa F., Uzair B. (2019). Fabrication of an original transparent PVA/gelatin hydrogel: In vitro antimicrobial activity against skin pathogens. Int. J. Polym. Sci..

[67] Pal K., Banthia A.K., Majumdar D.K. (2007). Preparation and characterization of polyvinyl alcohol-gelatin hydrogel membranes for biomedical applications. AAPS Pharm. Sci. Tech..

[68] Marrella A., Lagazzo A., Dellacasa E., Pasquini C., Finocchio E., Barberis F., Pastorino L., Giannoni P., Scaglione S. (2018). 3D porous gelatin/PVA hydrogel as meniscus substitute using alginate micro-particles as porogens. Polymers.

[69] Rizwan M., Yao Y., Gorbet M.B., Tse J.W., Anderson D.E.J., Hinds M.T., Yim E.K.F. (2020). One-Pot Covalent Grafting of Gelatin on Poly(Vinyl Alcohol) Hydrogel to Enhance Endothelialization and Hemocompatibility for Synthetic Vascular Graft Applications. ACS Appl. Bio Mater..

[70] Hui B., Zhang Y., Ye L. (2015). Structure of PVA/gelatin hydrogel beads and adsorption mechanism for advanced Pb(II) removal. J. Ind. Eng. Chem..

[71] Rodriguez-Abetxuko A., Sánchez-deAlcázar D., Muñumer P., Beloqui A. (2020). Tunable Polymeric Scaffolds for Enzyme Immobilization. Front. Bioeng. Biotechnol..

[72] Bermejo J.S., Ugarte C.M. (2009). Chemical crosslinking of PVA and prediction of material properties by means of fully atomistic MD simulations. Macromol. Theory Simul..

[73] Wu R., Qiu X., Yang X. (2016). Molecular dynamics simulations of atomistic hydration structures of poly(vinyl methyl ether). Chinese J. Polym. Sci..

[74] de Arenaza I.M., Meaurio E., Sarasu J.-R., De Souza Gomes A. (2012). Analysis of the Miscibility of Polymer Blends Through Molecular Dynamics Simulations. Polymerization.

[75] Li J., Jin S., Lan G., Chen S., Li L. (2018). Molecular dynamics simulations on miscibility, glass transition temperature and mechanical properties of PMMA/DBP binary system. J. Mol. Graph. Model..

[76] Wei Q., Wang Y., Che Y., Yang M., Li X., Zhang Y. (2017). Molecular mechanisms in compatibility and mechanical properties of Polyacrylamide/Polyvinyl alcohol blends. J. Mech. Behav. Biomed. Mater..

[77] Dong X., Liu Q., Cui L., Yu Y., Zhang M. (2014). Molecular simulation and experimental study on propylene dehumidification through a PVA-PAA blend membrane. J. Mater. Chem. A.

[78] Neffe A.T., Zaupa A., Pierce B.F., Hofmann D., Lendlein A. (2010). Knowledge-based tailoring of gelatin-based materials by functionalization with tyrosine-derived groups. Macromol. Rapid Commun..

[79] Knani D., Barkay-Olami H., Alperstein D., Zilberman M. (2017). Simulation of novel soy protein-based systems for tissue regeneration applications. Polym. Adv. Technol..

[80] Labus K., Drozd A., Trusek-Holownia A. (2016). Preparation and characterisation of gelatine hydrogels predisposed to use as matrices for effective immobilisation of biocatalystst. Chem. Pap..

[81] Lowry O.H., Rosebrough N.J., Farr A.L., Randall R.J. (1951). Protein measurement with the Folin phenol reagent. J. Biol. Chem..

[82] Abbott L.J., Hart K.E., Colina C.M. (2013). Polymatic: A generalized simulated polymerization algorithm for amorphous polymers. Theor. Chem. Acc..

[83] Zhao B., James M. (1999). Gelatin. Polymer Data Handbook.

[84] Shokuhfar A., Arab B. (2013). The effect of cross linking density on the mechanical properties and structure of the epoxy polymers: Molecular dynamics simulation. J. Mol. Model..

